# Complexins: small but capable

**DOI:** 10.1007/s00018-015-1998-8

**Published:** 2015-08-06

**Authors:** Ralf Mohrmann, Madhurima Dhara, Dieter Bruns

**Affiliations:** 1 Zentrum für Human- und Molekularbiologie, University of Saarland, CIPMM, 66421 Homburg/Saar, Germany; 2 Medical Faculty, Department of Physiology, University of Saarland, CIPMM, 66421 Homburg/Saar, Germany

**Keywords:** Complexin, Synaptotagmin, SNARE regulators, Membrane fusion, Ca^2+^ triggered exocytosis

## Abstract

Despite intensive research, it is still unclear how an immediate and profound acceleration of exocytosis is triggered by appropriate Ca^2+^-stimuli in presynaptic terminals. This is due to the fact that the molecular mechanisms of “docking” and “priming” reactions, which set up secretory vesicles to fuse at millisecond time scale, are extremely hard to study. Yet, driven by a fruitful combination of in vitro and in vivo analyses, our mechanistic understanding of Ca^2+^-triggered vesicle fusion has certainly advanced in the past few years. In this review, we aim to highlight recent progress and emerging views on the molecular mechanisms, by which constitutively forming SNAREpins are organized in functional, tightly regulated units for synchronized release. In particular, we will focus on the role of the small regulatory factor complexin whose function in Ca^2+^-dependent exocytosis has been controversially discussed for more than a decade. Special emphasis will also be laid on the functional relationship of complexin and synaptotagmin, as both proteins possibly act as allies and/or antagonists to govern SNARE-mediated exocytosis.

## Introduction

The Ca^2+^-triggered exocytosis of neurotransmitters and hormones is a tightly controlled process that has evolved to meet temporal precision and speed of intercellular communication. The core membrane fusion machinery is constituted by a set of three highly conserved proteins known as the SNAREs (*N*-ethylmaleimide-sensitive factor (NSF) attachment protein receptors) (for review see [1–3]). The vesicular SNARE protein synaptobrevin II (sybII) associates with its cognate target SNAREs, SNAP25 and syntaxin Ia (stxIa), on the plasma membrane to form a coiled-coil ‘SNAREpin’, crosslinking both membranes in the process. The formation of these membrane-bridging *trans*-SNARE complexes is believed to pull the lipid bilayers together and drive membrane merger, which finally unites the interacting SNAREs in *cis*-configuration on the fused membrane. SNARE proteins are characterized by SNARE domains of ∼60 amino acids, which form amphiphatic α-helices that can assemble into a thermodynamically stable coiled-coil helix bundle by favorable hydrophobic interactions of the inner helix faces and a number of salt bridges on the outside [4, 5]. The free energy of SNARE complex formation is used to overcome the strong repulsive force between both membranes and bring them into close apposition [6]. Complex formation is thought to start at the N-termini of SNARE proteins and progress in C-terminal direction in a zipper-like fashion [7–9]. Complete assembly of the SNARE complex is required to initiate efficient membrane merger. Although the three SNARE proteins are capable to induce slow fusion of liposomes in vitro, even in the absence of additional factors [10], Ca^2+^-dependent neurotransmitter release in vivo requires regulatory components that confer speed and precision to the fusion reaction [11]. Indeed, synapses in the mammalian brain typically possess an extensive set of accessory and regulatory factors, like, e.g., SM proteins, Munc-13, CAPS and synaptotagmin (syt) I, which seem to govern the fusion process through sequential mechanistic stages by regulating the assembly of the SNARE complex [12]. At active zones, vesicles rest in a “primed” fusion-competent state prior to Ca^2+^-triggered fusion and, therefore, SNARE assembly likely occurs in a discontinuous fashion allowing for a metastable fusion intermediate. Though alternative mechanistic scenarios have been discussed that conceive SNARE assembly as a one-step process downstream of triggering [13], detailed structure–function analyses (e.g., [9]) and biophysical assays probing the assembly of single SNARE complexes with optical tweezers delivered evidence for partially zippered intermediates that might be transiently stabilized by the repulsive forces between approaching membranes [14, 15]. However, one of the central open questions is how assembly of SNARE complexes is paused in a coordinated fashion to allow for fast synchronous release upon intracellular Ca^2+^-elevations. From a mechanistic perspective, the demanded metastable fusion intermediate might be upheld by the action of a SNARE-interacting protein that could serve as a transient fusion “clamp”. However, the existence and identity of the proclaimed “fusion clamping” factor have been debated for a long time.

### Complexins: a family of SNARE-interacting proteins

Complexins are likely the most controversially discussed SNARE-interacting proteins involved in exocytosis. As described in the course of this review, these small hydrophilic proteins (15–20 kDa) are suspected to play a major role in governing SNARE assembly during vesicle fusion. Complexins were first identified due to their ability to bind to and copurify with SNARE complexes [16, 17]. Today four different complexin genes, cplxI–cplxIV, have been described in mice, and corresponding orthologs also exist in the human genome [18]. CplxI and cplxII isoforms in mammalian species show an unusually high sequence conservation, which underlines their importance for regulated exocytosis. Indeed, the primary sequence of cplxII is identical in mouse, rat, and human, while cplxI still shows 97 % sequence conservation among murine and human orthologs [17, 18]. CplxI and cplxII are closely related isoforms (86 % sequence identity), but show only limited homology (24–28 % identity) to cplxIII and cplxIV, which seem to form a second subfamily [18]. Interestingly, the cplxI/II and cplxIII/IV subgroups mainly differ in their C-terminal domain, which—in the case of cplxIII/IV—carries an extension with a CAAX box motif for lipidation at its C-terminal end [18]. All four complexin isoforms are predominantly expressed in the central nervous system [16–18], with cplxIV protein being largely restricted to retinal ribbon synapses [18]. Complexin orthologs have also been identified throughout the animal kingdom, which suggest conserved function in regulated exocytosis. Interestingly, compared to mammals, invertebrates like *Caenorhabditis elegans* or *Drosophila melanogaster* express only a smaller number of complexin isoforms, which are sequence-wise closely related to the cplxI/II subfamily but frequently also contain a C-terminal extension with a CAAX farnesylation motif like cplxIII/IV [18, 19]. Thus, complexin isoforms in higher vertebrates likely evolved as functionally specialized versions of an ancestral protein fulfilling a more general role.

### Structural determinants of complexin

Complexins bind to the SNARE complex via an α-helical motif that is located near the center of the protein [17, 20, 21]. Of all known isoforms, cplxIV exhibits the lowest affinity for the SNARE complex, and thus efficient binding of cplxIV to the membrane-anchored SNARE complex critically depends on its correct localization at the plasma membrane via a farnesyl-anchor [18]. As recently shown by single molecule FRET experiments, cplxI not only binds to the ternary SNARE complex but also interacts with a 1:1 SNAP-25:stx1a complex [22], which might help to stabilize the putative acceptor complex during early stages of the fusion mechanism. Biochemical work by Jahn and coworkers [20] suggested that cplxI/II’s binding efficiency to the SNARE complex is determined by the identity of the SNARE isoforms incorporated in the target complex. Moreover, cplxI/II binding to the SNARE complex is very fast and occurs with high affinity [23–25]. Deuterium exchange experiments indicated that cplxI may stabilize the SNARE complex conformation, especially the assembled C-terminal region [21]. CplxII binding to the SNARE complex may also intensify interactions between the transmembrane regions of syntaxin and synaptobrevin [26].

**Fig. 1:**
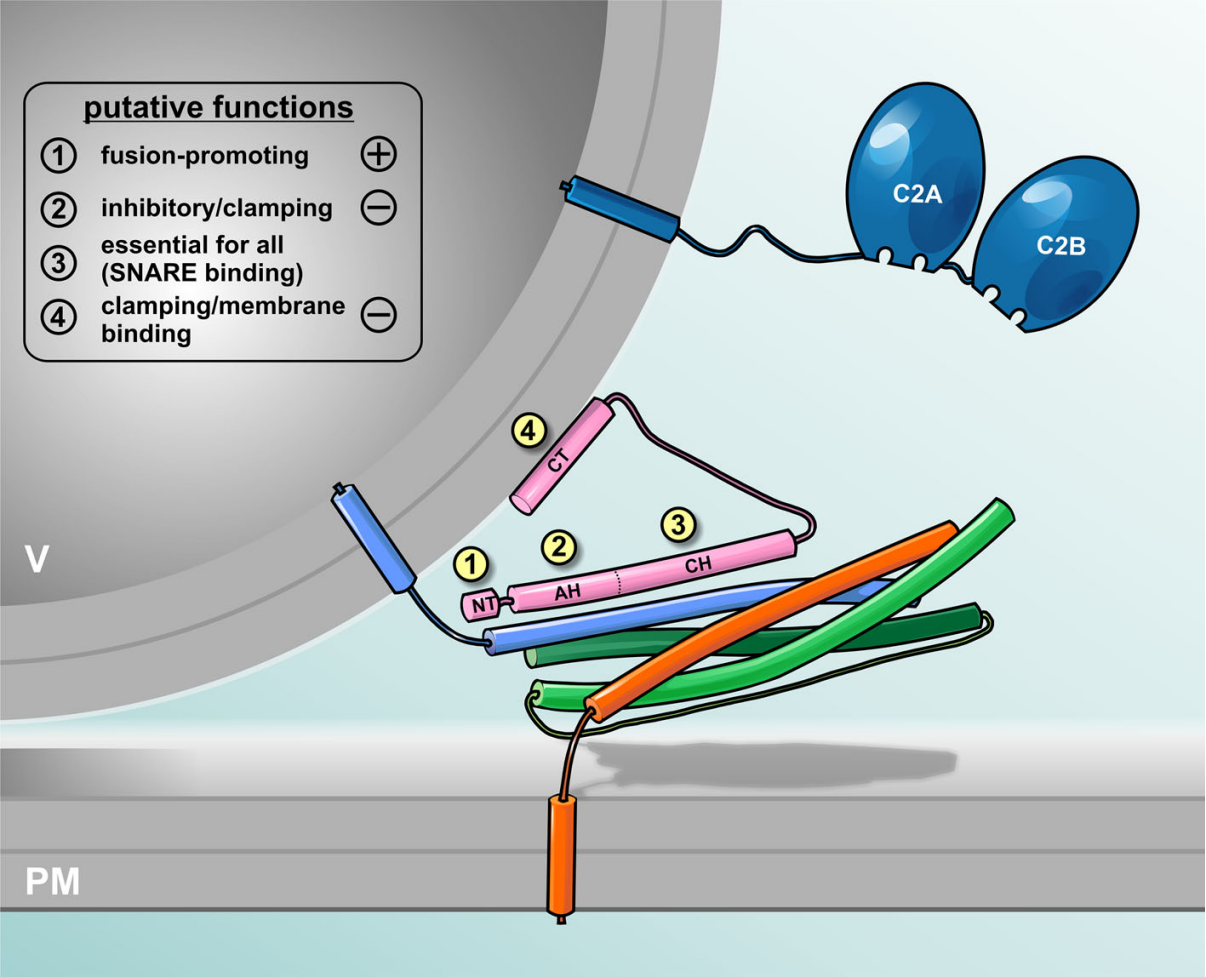
Hypothetical view on complexin and its interaction with the membrane-bridging SNARE complex. Vesicular SNARE (sybII, *blue*) and target SNARE (syx, orange and SNAP-25, *green*) partially assemble into trans-SNARE complex forming a high affinity binding site for complexin (*pink*). The N-terminus of Complexin (NT, amino acid 1–26) enhances fusion kinetics and fusogenicity [28, 45, 47, 48, 52, 63, 77, 78] while the accessory α-helix (AH, amino acid 27–47) [29, 41, 48, 49, 65–68, 71] and the C-terminus (CT, amino acid 73–134) [19, 34, 44, 50, 52, 63, 72] clamp premature release. The central helix (CH) of complexin binds with the SNARE complex [17, 20, 21, 27] which is prerequisite for all complexin actions [89]. The major Ca^2+^ sensor sytI (*blue*) interacts with SNAREs and membranes upon Ca^2+^-binding to its C2 domains, but is displayed separately for clarity of presentation

Complexin:SNARE complex interactions have been structurally resolved on atomic scale by X-ray crystallography demonstrating that an α-helical complexin fragment can attach in anti-parallel orientation to the groove formed between syntaxin and synaptobrevin [21, 27]. Amino acids 48–70 (rat cplxI) form the so-called ‘central helix’ in the middle of complexin, which constitutes the main binding interface ([21, 27], Fig. 1). Mutations of amino acids within this region diminish association of complexin with the SNARE complex [28]. The N-terminal region directly preceding the central helix (residues 29–47) seems to also assume a helical conformation [20, 21, 27, 29], and the motif has accordingly been named ‘accessory helix’ (Fig. 1). While this motif is not essential for SNARE binding, N-terminally flanking residues (amino acids 41–47) seem to enhance SNARE binding of the central helix [28]. Intriguingly, it has been postulated that helix formation is nucleated in the accessory helix and subsequently propagates into the region of the central helix, thereby potentially stabilizing the central helix and increasing SNARE binding [29]. Flanking sequences on the C-terminal side (residues 71–77) have also been suspected to contribute to the stabilization of the central helix [30]. Furthermore, in vitro phosphorylation of cplxI/II (Ser^115^) by protein kinase CK2 has been shown to strengthen complexin binding to ternary SNARE complexes, suggesting that complexin:SNARE interactions may be dynamically regulated by phosphorylation [31]. While complexin phosphorylation was demonstrated to occur in vivo at two sites [31, 32], it is currently unclear how phosphorylation of serine residues in the C-terminal domain could mechanistically influence the binding activity of the central helix.

Little is currently known about the structural features of the very N-terminus (residues 1–25) and the C-terminal domain (residues >83) of cplxI/II (Fig. 1). In the complexin:SNARE complex, these regions remain susceptible to proteolytic degradation and do not assume an α-helical conformation [20]. This suggests that they do not engage in tight interactions and may possibly stay unstructured. Recent studies, however, indicated that the C-terminal domain may contain an amphipathic helix that could bind to phospholipids [33, 34] and thus might contribute to localize complexin to synaptic vesicles. Such membrane-anchoring function of the C-terminus of complexin would be in line with the existence of prenylation sites in multiple invertebrate complexin isoforms as well as cplxII/IV in mammals. The sole *D. melanogaster* ortholog of complexin was recently shown to possess two C-terminal splice variants, of which one lacks the CAAX-box required for prenylation [19]. In addition, the C-terminal domain of complexin is subject to mRNA-editing further modifying its sequence. These findings support the notion of a functionally relevant specialization of the C-terminal domain in different isoforms.

### Function of complexin: to clamp or not to clamp?

Fast Ca^2+^-regulated exocytosis in secretory cells relies on a functionally distinct pool of primed vesicles, which are ready to fuse in response to a triggering Ca^2+^-stimulus. A second larger pool of morphologically docked vesicles, which is typically referred to as ‘depot pool’, serves as a replenishing reservoir to compensate for vesicle loss by exocytosis. For both types of vesicles, the dimensions of intermembrane distances are compatible with membrane-bridging interactions of SNARE proteins [35, 36] raising the possibility that SNAREs assemble spontaneously [9, 10, 37] and cause premature loss of vesicles. The untimely fusion of vesicles may contribute to so-called spontaneous release, a form of untriggered vesicle fusion occurring alongside evoked synaptic transmission at most chemical synapses. While the specific role and the regulation of the spontaneous release component are not fully understood (for a recent review see [38]), it is clear that the majority of docked vesicles is reluctant to fuse with the plasma membrane in the absence of a proper stimulus. Hence, a molecular mechanism must exist that effectively arrests vesicles in the docked state allowing for an appropriate stimulus-secretion coupling. Although other mechanisms like restricted v-SNARE accessibility [39] might contribute to the attenuation of premature release, complexin has been proposed to play the principal role in “clamping” primed vesicles.

Initial in vitro analyses using a liposome fusion assay [40] or Hela cells that ectopically express “flipped” SNAREs on their cell surface [41] showed that complexin can inhibit the SNARE-driven fusion machinery providing direct evidence for a negative modulatory role in exocytosis. In close correlation, genetic ablation of the relevant complexin isoforms in the NMJs of invertebrates leads to a strong increase in spontaneous release [34, 42–45]. In contrast, knock-out and knock-down perturbations of murine complexin resulted in opposing views about its role in exocytosis. While genetic ablation of all complexin isoforms expressed in brain either does not alter or even reduces spontaneous release in autaptic hippocampal cultures [28, 46] and brain slices [47], knockdown of cplxI/II by RNA interference in mass cultured cortical neurons increases spontaneous release [48–50]. More recent experiments in mass cultured cortical neurons, designed to deconstruct these phenotype differences, have shown that genetic loss of cplxI/II unclamps spontaneous release [51]. Yet, in the same study, it has been reported that knock-down of cplxI/II leads to complementary overexpression of cplxIII and cplxIV. Since cplxIII expression in wild-type cells enhances spontaneous release, it remains to be clarified to what extent the unclamping phenotype is due to loss of cplxI/II or off-target effects on cplxIII expression. CplxII knock-out in chromaffin cells also demonstrated an enhanced tonic release which is evident at elevated levels of [Ca]i (>100 nM), but absent at low resting [Ca]i [52]. Given this observation, it is tempting to speculate that variations in [Ca]i among the different preparations may contribute to the deviating expression of the complexin null phenotype in different preparations.

In the same line, several studies boosting complexin action by either genetic overexpression or peptide supplementation have provided evidence for the complexin clamp function in neuronal and non-neuronal cells. Expression of either cplxI or cplxII markedly suppresses acetylcholine release from PC12 cells [53, 54] and also strongly impairs hGH secretion from insulin secreting cell lines [55]. Overexpression of cplxII in bovine [56] and mouse chromaffin cells [52] also reduces catecholamine secretion. Elevating local concentration of cplxI via a cplxI-sybII fusion protein that selectively expresses at the synapses of wild-type murine neurons impairs spontaneous synaptic vesicle fusion [57]. Moreover, acute dialysis of zebrafish or mouse retinal bipolar cells with a peptide derived from the conserved SNARE-binding domain of cplxIII/IV increases spontaneous release, most likely by competing with endogenous complexin for SNARE binding [58, 59]. In acrosomal exocytosis, supplementing permeabilized human sperm cells with cplxII arrests exocytosis by clamping a loosely assembled *trans*-SNARE complex [60].

Based on these findings, it stands to reason that loss of the complexin clamp action, particularly at elevated resting [Ca]i, should lead to a depletion of the vesicle pools due to unfettered exocytosis. Indeed, ultrastructural and high-resolution imaging studies have shown that loss of complexin results in a specific loss of membrane proximal vesicles at *C. elegans* NMJ [43] and mouse chromaffin cells [52]. Likewise, in absence of the complexin clamp function, the depot pool of synaptic vesicles is also depleted in the zebrafish and mouse retinal bipolar cells [58, 59]. Conversely, autaptic hippocampal preparations that do not show any increase in spontaneous activity in the absence of cplxI and cplxII also reveal no change in vesicle docking [46], a phenotype recently confirmed with state-of-the art EM tomography of synaptic structures in hippocampal brain slices [61]. In vitro liposome fusion assays also display a decreased vesicle association that is accompanied with enhanced spontaneous fusion in the absence of complexin, emphasizing its clamp role [62, 63]. That said, it should be noted that complexin has been shown to increase the on-rate of docking in liposome fusion assays [64]. In contrast, loss of complexin in *Drosophila* massively increases spontaneous release but neither affects the number of total nor of docked SVs at the NMJ [42, 45]. Given such excessive release in the absence of complexin, one might speculate that mechanisms of the insect NMJ have specially adapted to perpetuate the high rate of vesicle exocytosis by speeding-up replenishment reactions, masking potential vesicle depletion.

In conclusion, despite some remaining uncertainties, the combined set of data from in vitro and in vivo studies provides a model where complexin takes center stage in clamping of premature vesicle release.

### Mechanism of complexin’s clamp function

How does complexin clamp premature exocytosis? In vitro analyses in Hela cells by Rothman and colleagues demarcated a region comprising amino acids 26–83 of cplxI as the ‘minimal clamping domain’ of the protein. According to their comprehensive mechanistic model, binding of the complexin central helix (amino acids 48–70) to the SNARE complex is a prerequisite for protein function, and interaction of the complexin accessory α-helix (amino acids 26–47) with the partly zippered SNARE complex inhibits complete C-terminal assembly and membrane fusion. The accessory helix is thought to compete with the C-terminal portion of sybII for binding to its cognate SNARE partners, hence providing an on–off switch by alternative zippering [41, 65]. This mechanistic idea is based on sequence similarities between the sybII hydrophobic layers (layer position +3, +4 and +7) and the accessory helix of complexin (aligned in antiparallel orientation) and was further tested by generation of complexin mutants with enhanced sequence similarities (sybII-mimetic, ‘superclamp’ mutation) or with sequence modifications putatively decreasing this interaction (sybII-divergent, ‘poor clamp’ mutation), which should facilitate or hinder alternative zippering and thus modulate clamping activity [65]. Whereas in vitro fusion studies using these mutants delivered the expected results for clamping [65], and binding assays showed corresponding small changes in affinity to *cis*-SNARE complexes [49], in vivo studies attempting to rescue the knock-down or knock-out phenotype revealed inconsistent results regarding the efficacy of the mutant proteins to either superclamp (sybII-mimetic mutation) or unclamp (sybII-divergent mutation) spontaneous release [49, 66, 67]. This illustrates some mechanistic differences in the action of complexin in a physiological context and in reductionist assays like cell–cell fusion.

Kümmel et al. recently addressed the structural configuration of the complexin-clamped prefusion SNAREpin by studying a complex formed between the cplxI superclamp mutant and a SNARE complex containing a C-terminally truncated sybII variant, in which the accessory helix of complexin can stably zipper into the complex without interference of the competing region of sybII [68]. Intriguingly, the crystal structure of this complex suggested a variation of the original model, wherein the central helix of complexin binds to one SNARE complex, while the adjacent accessory helix binds to a neighboring, second SNARE complex [68, 69]. Based on these results, it has been suggested that complexin may organize SNARE complexes into a zigzag array that—when interposed between vesicle and plasma membranes—hinders fusion. Yet, the general hypothesis of insertion of the accessory α-helix into the partially assembled SNARE complex (either within or between complexes) is still highly controversial due to conflicting results of ITC, FRET, and NMR analyses addressing the underlying interactions between accessory α-helix and SNARE bundle [67, 70]. It remains to be seen, whether future studies can conclusively confirm this model.

Interestingly, Trimbuch et al. demonstrated a tenfold decrease in the binding affinity of complexin’s central helix to the SNARE complex upon truncation of the accessory α-helix. This suggests an indirect effect of this motif on complexin:SNARE interactions—a notion that agrees with biochemical experiments showing decreased complexin binding to the SNARE complex in absence of the accessory α-helix [28]. Thus, it is possible that helicity of this region is crucial for stabilizing complexin binding to SNAREs. Based on the concentration of negatively charged amino acids within the accessory α-helix, Trimbuch and colleagues [67] posited a model, wherein this protein region inhibits release through enhancing electrostatic repulsion between vesicle and plasma membranes. Nevertheless, it remains to be shown to what extent substitution or addition of negatively charged amino acids alter the domain’s helicity or the overall binding affinity for the SNARE complex. Another molecular mechanism for the accessory α-helix mediated clamp action has recently been proposed by Bykhovskaia et al. [71]: using molecular dynamics simulation, they concluded that the accessory α-helix interacts directly with the v-SNARE sybII and thus arrests the zippering of the last hydrophobic layers +7 and +8. In this context, it is important to note that recent experiments at the NMJ in *C. elegans* have shown an impaired complexin inhibition, if helix propagation into the central helix of complexin was disrupted [29]. Astonishingly, even replacing the accessory α-helix with a non-native helical sequence restored complexin function, suggesting that neither primary protein sequence nor hydrophobicity or net charge density of the accessory α-helix is required for complexin inhibition. Yet, another mode of accessory α-helix-mediated clamping action has been proposed for murine central synapses wherein the accessory α-helix putatively clamps an unidentified secondary Ca^2+^-sensor whose activation would cause unfettered vesicle fusion in absence of complexin [49].

**Fig. 2:**
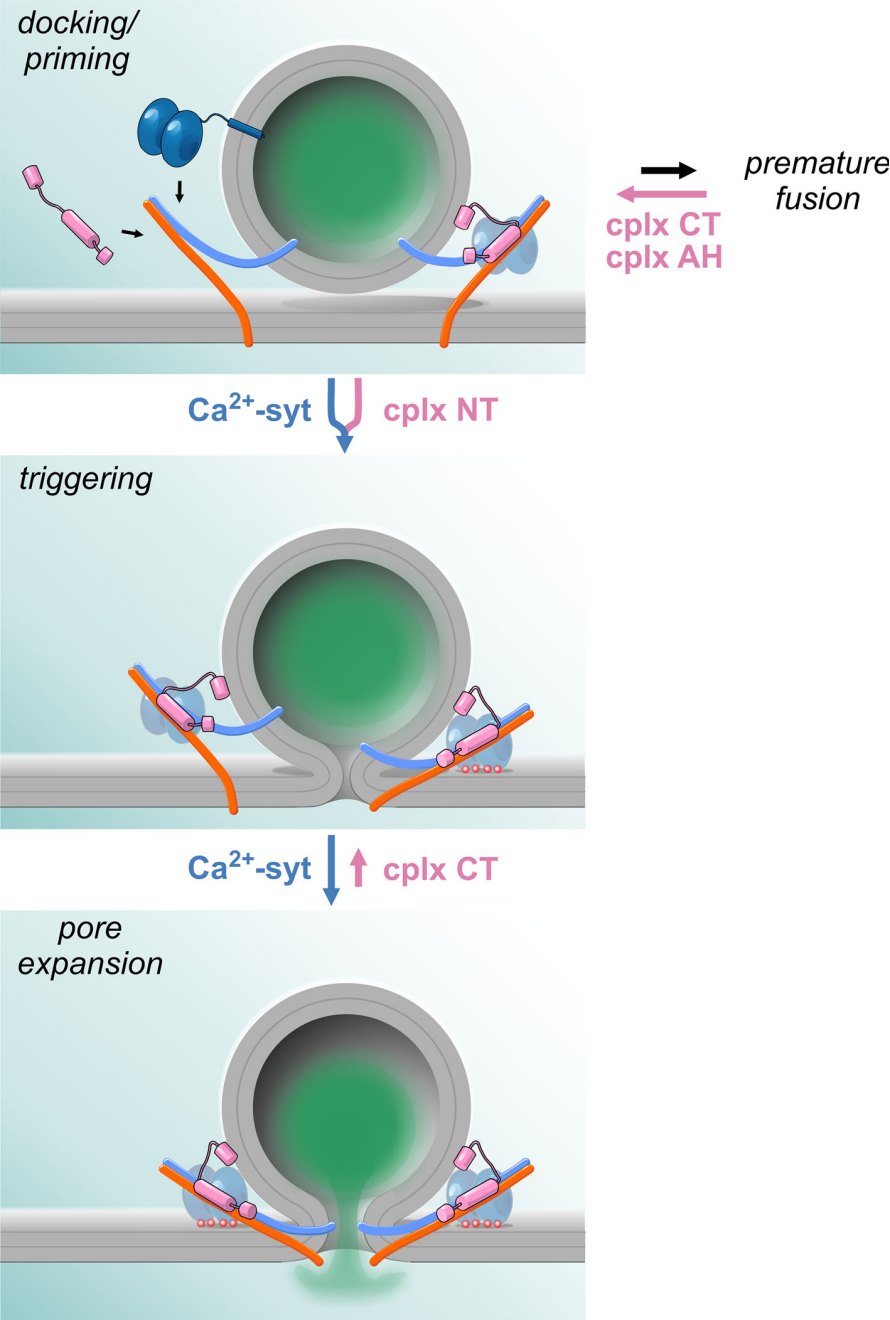
Hypothetical model of complexin action on various steps leading to vesicle exocytosis. Spontaneous SNARE zippering may lead to premature fusion of docked or primed vesicles in the course of vesicle maturation. Complexin with its accessory α-helix and C-terminus prevents the premature loss and thereby increases the pool of primed vesicles. Furthermore, N-terminus of complexin accelerates the kinetics of primed vesicle fusion serving as an ally of sytI in synchronizing the release response. Therefore, complexin promotes synchronous vesicle fusion by two distinct but synergistic functions. The clamp action of complexin C-terminus is continued from ‘docking’ until fusion ‘triggering’ where Ca^2+^-bound sytI effectively antagonizes the clamp leading to rapid fusion pore expansion

Evidently, despite a large amount of experimental efforts and various possible hypotheses, a satisfying consensus regarding the mechanism by which the accessory α-helix may clamp premature release has not been reached. However, recent in vitro and in vivo experiments have indicated that the C-terminus (amino acid 72–134) of complexin can also exert a fusion clamping function (Figs. 1, 2). Once considered to be functionally inert [28], later experiments have shown that the C-terminal domain actively clamps spontaneous liposome fusion as well as synaptic vesicle exocytosis in both invertebrate [19, 34, 44, 72] and vertebrate neuronal preparations [50]. Furthermore, experiments at the NMJ of *C. elegans* suggested that the C-terminal domain of complexin tethers the protein via its amphipathic helix to synaptic vesicles and thus concentrates the SNARE-binding region at the site of exocytosis for efficient clamping [34]. However, experiments in chromaffin cells counter the hypothesis of a simple targeting role of the complexin C-terminus. They show that a C-terminal truncation mutant (amino acids 1–72) actively ‘unclamps’ tonic secretion with expression in wild-type cells [52]. These observations indicate that the mutant competes with endogenous complexin for binding to productive SNARE complexes, but has lost its ability to clamp tonic secretion. Thus, the C-terminus actively suppresses premature exocytosis, a property that may also rely on lipid binding of this protein domain [33, 34, 50, 73]. Given that two independent domains of complexin have been shown to clamp spontaneous exocytosis, an attractive hypothesis could be that the C-terminus actually folds back onto the accessory α-helix, where it may promote protein–lipid and protein–protein interactions with its amphipathic helix. Such interactions may then stabilize the position of the accessory α-helix on the SNARE complex. Undoubtedly, more experiments addressing the mechanistic function of the C-terminus are required for a true understanding of the physiological clamp role of complexin.

### Two in one sweep: facilitation of fusion as a secondary function?

Knock-out and knock-down studies of complexin have shown, as a common denominator, a prominent reduction of evoked release, likely pointing to a direct facilitatory role of complexin in synchronous neurotransmitter release [28, 42–52, 57, 72, 74–79]. While compromised evoked release may be due to depletion of primed vesicles by premature spontaneous fusion [42, 45, 52, 58, 59], this explanation cannot be generalized for all types of preparations. In model systems, in which spontaneous fusion rate is unaffected by the abolishment of complexin, like, e.g., in autaptic microisland cultures, diminished evoked release has primarily been explained by a lowered release probability rather than a loss of primed vesicles [28, 46, 77].

In cultured neurons, the number of highly primed synaptic vesicles, which rapidly undergo exocytosis upon a Ca^2+^-stimulus and thus are thought to form a so-called ‘readily releasable pool’ (RRP), can be directly estimated by application of hypertonic solution (500 mM sucrose). It is believed that this method induces Ca^2+^-independent release by subjecting synapses to an osmotic shock, possibly forcing vesicle fusion by mechanical stress. Intriguingly, this technique did not reveal any reduction in pool size for complexin-deficient hippocampal neurons in autaptic microisland cultures [28, 46, 77], which largely excludes vesicle depletion as the cause of compromised synaptic transmission. Rather, Xue et al. [77] noticed a slightly delayed release kinetic upon hypertonic challenge in complexin-deficient synapses, which argues in favor of a reduced fusogenicity of RRP vesicles in the absence of complexin. In addition, a milder hypertonic shock (250 mM sucrose) that does not fully deplete the RRP was less efficient in inducing release in complexin-deficient cells than in wild-type controls, which again indicates that vesicles reside in a more fusion-reluctant state after ablation of complexin [77]. Thus, deficits in evoked release must be predominantly caused by the loss of a fusion-facilitating effect of complexin in microisland cultures. Interestingly, in the case of cultured cplxII^−/−^ chromaffin cells, in which premature release clearly diminishes the built-up of primed vesicle pools, an additional reduction in vesicular release rates and a significant delay in secretion onset have been observed in response to a step-wise increase in [Ca]i [52]. These observations also agree with the notion of a faltering fusion rate of primed vesicles in the absence of complexin. Moreover, intriguing kinetic changes of action potential evoked synaptic responses have been demonstrated in several preparations: at fly and murine NMJs, the genetic ablation of complexin results in a desynchronization of release [45, 78, 80], which noticeably broadens the waveforms of evoked synaptic responses, while the kinetics of synaptic miniature events remains unchanged [45, 78]. These waveform changes likely reflect a delayed and scattered release of individual quanta, once again pointing to impeded fusion of primed vesicles in synapses lacking complexin. Furthermore, single-vesicle content mixing and liposome fusion assays have provided convincing evidence for an enhanced Ca^2+^-control of vesicle fusion in the presence of complexin [62, 81–83].

Thus, phenotypic cues from the vast majority of model systems as well as in vitro analyses indicate a fusion-promoting action of complexin that either complements concurrent complexin-mediated “clamping” of spontaneous fusion or even represents its chief function depending on the particular model system.

### Mechanistic insights into the fusion-promoting function of complexin

For a true understanding of the role of complexin in transmitter release, it is of utmost importance to elucidate the exact mechanism underlying its facilitatory function and to clarify whether facilitation is mechanistically independent of clamping. To this end, it is helpful to review available cues on the identity of complexin domains involved in fusion-facilitation and to discuss their potential mechanistic function. Employing the microisland culture system, Xue et al. [28] found that the N-terminal region (residues 1–26) is required to fully rescue evoked release in cplxI^−/−^ hippocampal neurons (Fig. 2). Interestingly, mutation of residues 3–6 in cplxI eliminates the facilitating effect on evoked release and impairs rescue in knock-out neurons [77]. Based on structural considerations and biophysical experiments, Rosenmund and coworkers further concluded that the involved N-terminal motif forms an amphiphatic α-helical segment that binds to the C-terminal end of the SNARE complex. Consequently, the observed facilitating effect on evoked release may be explained by the binding of this helical motif to the SNARE complex, which could provide conformational support to the assembling C-terminus during final stages of exocytosis. In accord with this, Südhof and colleagues [48] reported that a truncated complexin variant (residues 27–134) is unable to rescue evoked release after complexin knock-down but still reconstituted normal spontaneous release. Due to related phenotypic features of synaptobrevin linker mutants (sybII W^89^A, W^90^A; but cf. [84]), this study proposed that the complexin N-terminus is somehow assisting mechanical force transfer onto membranes.

Noteworthy, a reduction of Ca^2+^-sensitivity of evoked release has indeed been reported for complexin-deficient neurons/endocrine cells in most preparations [28, 42, 46, 52, 85] (but see [49]), which rather supports the idea of a mechanistic crosstalk between complexin and the Ca^2+^-sensor synaptotagmin. Furthermore, some studies [45, 85] have been able to observe a clear decrease in the Hill coefficient for the Ca^2+^-cooperativity of release in complexin-deficient cells. In the same line, expression of an N-terminally truncated complexin variant (residues 28–134) in cplxII^−/−^ chromaffin cells failed to re-establish normal release rates, prolonged the secretory delay and lowered the apparent Ca^2+^-affinity of secretion [52]. Exocytosis timing in chromaffin cells is largely determined by the kinetics of Ca^2+^-binding to sytI [86]. Thus, the mutant properties are characteristic for a decreased forward rate of Ca^2+^-binding to the calcium sensor, hence, pointing again to a role of the complexin N-terminus in modulating sytI function (Fig. 2). Apart from kinetic changes, Dhara et al. [52] reported that the N-terminally truncated complexin variant could largely restore the overall amplitude of Ca^2+^-triggered secretion in cplxII-deficient chromaffin cells—seemingly in contrast to diminished evoked release in neurons using similar mutants [28, 77]. However, the data might be easily reconciled, when considering the different durations of triggering Ca^2+^-stimuli used in these preparations. Under conditions of sluggish stimulus secretion coupling, chromaffin cells can still empty the entire primed vesicle pool due to the long-lasting Ca^2+^-stimulus. In neurons, however, slow stimulus-secretion coupling in response to a rapid action potential evoked Ca^2+^-transient would certainly cause a significant drop in the EPSC amplitude, providing an attractive explanation for the facilitatory phenotype of complexin’s N-terminus as well as for kinetic changes of endplate responses at the NMJs of complexin null mutants [45, 78]. Nevertheless, by comparing the phenotypes of single null mutants for complexin and sytI, cplx^−/−^; sytI^−/−^ double-deficiency and overexpression experiments, additive as well as interdependent effects on release probability and exocytosis timing have been observed in hippocampal neurons and the NMJ of Drosophila [45, 77], leaving the exact mechanistic relationship between both proteins unclear.

Work by the Südhof group has recently added another intriguing facet to the putative facilitation mechanism by proposing that complexin may also play a major role in vesicle priming. This conclusion was reached mainly based on cplxI/cplxII knock-down experiments in cortical mass cultures [49–51]. In contrast to the release phenotype found in microisland cultures (e.g., [28, 46]), knock-down or knock-out of complexin in this type of neuronal preparation resulted in a substantial increase in spontaneous release as well as a strongly reduced RRP size, as assayed by hypertonic challenge. Intriguingly, Kaeser-Woo et al. [50] demonstrated that a C-terminally truncated variant (residues 1–86) can rescue evoked release but does neither re-establish normal RRP size nor diminish elevated spontaneous release. While these results confirm that the N-terminal domain is needed to sustain effective triggering, they also suggest that the C-terminal domain is required for efficient priming besides fusion clamping, consequently attributing the overall facilitatory function of complexin to independent actions of its two subdomains. This being said, it is obviously troublesome that the phenotypic hallmarks of the suspected complexin-dependent priming mechanism are not equally well recognizable in all preparations—even in so closely related culture types. Furthermore, recent tomographic EM analyses were able to present some morphologic correlates for priming by studying SNARE-deficient synapses in hippocampal slice culture, but were unable to pinpoint a morphological priming defect in complexin-deficient neurons [61]. Possibly, the priming function of complexin is a non-essential feature that can be compensated for by redundant mechanisms.

The idea of a facilitatory role of the N-terminal complexin motif was lately also confirmed in trans-species rescue experiments, in which a complexin chimera that contains the N-terminus (residues 1–16) of *D. melanogaster* and complementary sequences from the murine ortholog (residues 17–134) was able to reconstitute normal evoked release in murine complexin-deficient neurons [76]. However, the interpretation of such experiments is complicated by the fact that fly and murine complexin orthologs only possess limited functional interchangeability. Indeed, full-length fly complexin is unable to substitute for murine isoforms in rescue experiments and even suppresses synaptic release when expressed in mouse wild-type neurons, while expression of murine cplxI-III in *D. melanogaster* overly increases evoked synaptic responses [72, 76]. Contrary to previous findings in mammals, Cho et al. [66] found that expression of an N-terminally truncated murine complexin variant (residues 51–134) was fully able to rescue evoked transmission when expressed in *Drosophila* null mutants, which challenges the view of the fusion-promoting function of the N-terminal motif. Likewise, N-terminally truncated variants of the *C. elegans* ortholog seem to completely rescue evoked release at body wall-muscle NMJs [43, 44]. Moreover, the truncated complexin variant (residues 16–143) tested by Hobson et al. [43] not only increased the amplitude of evoked EPSCs over the level of wild-type controls but also promoted spontaneous release in the absence of extracellular Ca^2+^—thus basically inverting the functional assignment of domains established in mammals. It is currently not clear, how to reconcile these contradicting findings in vertebrates and invertebrates, since the N-terminal region of *D. melanogaster* and *C. elegans* complexin shows some sequence homology with murine cplxI/II and, thus, mechanistic similarities could be expected. One possible explanation for this dilemma might be seen in the specialized functional properties of invertebrate neuromuscular junctions that set them apart from central synapses found in the central nervous system of vertebrates. In particular, the *C. elegans* NMJ is unique with respect to its high rates of spontaneous release (around 50 Hz). The physiological function of this high spontaneous synaptic activity is still unknown [87], but it might be speculated that the release machinery at these synapses evolutionary adapted to generate a specialized pattern of synaptic activity. Following this idea, the mechanistic role of complexin may also have changed during the evolutionary adaptation of NMJ physiology, possibly by tweaking its interaction with other factors governing SNARE assembly.

To test the mechanistic function of specific complexin domains under well-defined conditions in vitro, Lai et al. [63] recently used a single liposome–liposome content mixing assay and quantified liposome association, spontaneous fusion, amount of Ca^2+^-triggered fusion, and synchronization of Ca^2+^-induced release. In good correlation with in vivo analyses in vertebrates, they found that Ca^2+^-induced fusion events in this model system occurred less frequently and less synchronized in the presence of complexin mutants lacking the N-terminus (amino acid 27–134), while spontaneously occurring release before application of Ca^2+^ was only changed in the absence of the complexin C-terminus (amino acids 1–86). So, it can be even recognized in a strongly reduced system only containing the minimal fusion machinery that the complexin N-terminus is critically involved in enhancing the fidelity of liposome fusion.

In summary, there is increasing consensus that the major fusion-promoting function of complexin in vertebrates is mediated by its very N-terminus. This facilitatory action seems mechanistically independent and even separable from the clamping function of complexin, which is putatively mediated by the accessory α-helix together with the C-terminus (s. above). However, in invertebrates the mechanistic role of complexin domains may deviate from this pattern. Overall, these findings strengthen the view that complexin conveys two synergistic functions to enhance synchronous fusion of vesicles: (1) maintenance of a proper primed vesicle pool by preventing its premature depletion and (2) facilitation of fusion in response to the Ca^2+^-trigger.

### Synaptotagmin: ally and antagonist?

In previous sections, we have discussed the janus-faced actions of complexin during fusion—but have only marginally touched upon one mechanistic aspect that might actually help to tie both functions together, namely the interplay between complexin and the Ca^2+^-sensor sytI. Indeed, it is immediately evident that the postulated complexin-mediated ‘fusion clamp’ must be rapidly lifted when fusion is triggered by above-threshold Ca^2+^-transients and that the activation of the arrested state directly or indirectly depends on an antagonistic action of sytI. In addition, the facilitatory action of complexin seems to increase release probability and calcium sensitivity in the majority of preparations, making sytI again appear as a relevant interaction partner for complexin (Fig. 2). Thus, the mechanistic relationship between complexin and sytI is of central importance for our understanding of complexin function.

While there is a notable consensus that sytI promotes SNARE assembly and vesicle exocytosis upon presynaptic Ca^2+^-elevations (e.g., [88, 89]), major aspects of its molecular function have still remained enigmatic to date. Structurally, sytI is a transmembrane protein that contains two C2 homology domains, denoted C2A and C2B, within its cytosolic part. SytI binds Ca^2+^, phospholipids, and the SNARE complex via its C2 domains, though the specific binding configuration is not yet clear (for a detailed review see [90, 91]). Intriguingly, ablation of sytI leads to a complex secretion phenotype featuring a conspicuous desynchronization of release as well as an elevated rate of spontaneous fusion in some model systems [92–98] but not others [88, 99]. These observations are highly reminiscent of the controversial phenotypes found with complexin ablation. Given the inhibitory effects of sytI on spontaneous activity in several preparations, some studies have entertained the idea that sytI itself could act as a major component of the fusion clamp [100–102]. So, are sytI and complexin potential allies in suppressing premature release? While the idea of an sytI-mediated clamp mechanism has initially received support from in vitro studies demonstrating an inhibitory effect of the isolated sytI C2AB domain on liposome fusion in the absence of Ca^2+^ [103, 104], other studies indicated a general fusion-promoting function of the full-length protein arguing against genuine clamping by sytI in reduced model systems [62, 82, 83, 105–110]. Alternatively, the observed increase in spontaneous release rate in the absence of sytI could be explained by other syt isoforms improperly deputizing for the role of calcium sensor [96, 98]. That said, it should be noted that a GABAergic modulation of spontaneous glutamatergic release rate was recently shown to influence the expression of the sytI knock-out phenotype in some model systems [111]. In any case, a potential mechanistic connection between complexin and sytI should be most obvious in double knock-out mutants that are deficient for both sytI and complexin. Indeed, several groups have recently generated and tested such double knock-out mutants in mice and flies [45, 52, 77]. If both proteins would “clamp” release cooperatively or independently at the same mechanistic step, an unchanged or even exacerbated spontaneous release rate would be expected to occur in double mutants. Surprisingly, however, Jorquera et al. and Dhara et al. similarly reported that the phenotype of cplx^−/−^; sytI^−/−^ double mutants is virtually identical to the one seen in sytI single knock-outs and also abolishes the pronounced rate of spontaneous release typically observed in cplx^−/−^ flies and the increased tonic secretion in neuroendocrine cells. Thus, there clearly is a strong mechanistic interdependence between the actions of both proteins, but no mechanistically overlapping function in fusion clamping. Indeed, it has been speculated that Ca^2+^-independent binding of sytI to the SNARE complex may increase the propensity of the complex to zipper up and promote fusion. This inherent “leakiness” of the sensor-system under resting conditions might be countered by the action of complexin [45].

An antagonism between complexin and sytI also constitutes the very backbone of popular concepts explaining the relief of the complexin-mediated clamp of spontaneous release. Mainly based on experimental cues from in vitro fusion experiments [40, 41, 62, 65, 69, 112], it has been proposed that a complexin-stabilized fusion intermediate (see previous chapters) is activated by Ca^2+^-bound sytI leading to subsequent C-terminal assembly of the SNARE complex and membrane merger. Interestingly, biochemical work by the groups of Rizo and Südhof presented evidence for a mutual exclusive binding of both proteins to the SNARE complex and even demonstrated that either protein can expel the other when presented at high enough concentrations [57, 113]. These findings led to the mechanistic idea that sytI may antagonistically displace complexin from the SNARE complex in a Ca^2+^-dependent fashion and that this ‘complexin–synaptotagmin-switch’ may underlay fusion triggering. Nevertheless, the postulated competitive binding and displacement of complexin by sytI have been highly controversial due to contradictory biochemical results indicating a concurrent association of both proteins with the SNARE complex [114]. Another study by Tokumaru et al. [115] even postulated a C-terminal interaction of complexin with sytI and speculated that complexin might be involved in recruiting sytI to the SNARE complex—basically inverting the ‘complexin–synaptotagmin-switch’-idea. Reconciling some of the experimental controversies, Rizo’s group revealed in a recent study that competitive effects between sytI and complexin might be more subtle than previously assumed (possibly restricted to subdomains) and depend on the experimental conditions, especially whether or not the SNARE complex is in a membrane-attached state [116]. Moreover, single-molecule FRET studies to elucidate the sytI:SNARE binding configuration have led to a model predicting largely unobstructed complexin binding to the groove formed by syntaxin-1A and synaptobrevin even when C2AB is simultaneously attached [117]. Direct evidence for a persistent binding of complexin to the SNARE complex has also come from total internal reflection fluorescence microscopy experiments, in which GFP-tagged complexin was shown to be recruited to prospective fusion sites, remained at this position until after fusion, and was eventually diminished by lateral spreading in the membrane [118]—which strongly indicates that complexin remains attached to the *cis*-complex after membrane merger. Thus, there is now accumulating evidence that both proteins can bind simultaneously in a non-overlapping configuration to the SNARE complex, and that the antagonism of both proteins does not result from competition for binding sites.

If Ca^2+^/sytI-mediated complexin displacement from the SNARE complex represents an unlikely mechanism, how else can the antagonistic function between sytI and complexin be envisioned? Some intriguing observations have lately been made in chromaffin cells, in which the catecholamine release from single secretory granules can be studied by amperometric recordings. Using this technique, it could be shown that sytI loss delays the initial fusion pore dilation and that this phenotype was reversed by additional elimination of cplxII in sytI^−/−^; cplxII^−/−^ double knock-out cells [52]. These results suggest that the prolonged fusion pore dilation seen in sytI-deficient cells is actually caused by an action of cplxII, in accord with earlier studies showing that complexin can impact fusion pore behavior [56, 118]. This interpretation was further supported by the finding that overexpression of cplxII in wild-type cells could mimic the secretion phenotype of sytI^−/−^ cells, illustrating an antagonistic action of both proteins in controlling early fusion pore dynamics [52]. Although cplxII deficiency was shown to have no effect on fusion pore dilation at high Ca^2+^ (20 µM) in this study, it increasingly shortens the initial fusion pore duration with lowering [Ca]i. This implies a push–pull mechanism, wherein a sytI/Ca^2+^-mediated acceleration of fusion pore dilation counteracts a cplxII ‘clamp’ action that counters pore expansion. Intriguingly, the C-terminal domain of complexin that is involved in suppressing premature fusion was found to be also responsible for clamping the fusion pore. Thus, sytI in its Ca^2+^-bound form overcomes cplxII-mediated restraints on force transduction at the moment of the Ca^2+^-rise to initiate formation and accelerate dilation of fusion pore—putting both proteins in a truly antagonistic relationship during the last steps of exocytosis. On the molecular level, the functional antagonism between complexin and sytI may be due to the induction of limited conformational changes upon Ca^2+^-binding, as both factors likely reside on the SNARE complex.

Though little is currently known about the conformational states involved in triggering, existing cues might at least provide welcome primers for future research avenues: Krishnakumar et al. [69] recently employed FRET experiments to investigate conformational changes of the complexin accessory helix during SNARE zippering and reported that the accessory helix converges onto the SNARE complex during assembly of the final layers. Intriguingly, the authors conclude that switching to a ‘closed’ conformation is required for fusion triggering, as a synaptobrevin variant that fails to undergo the conformational transition due to mutation of a group of residues (syb D^64^A, D^65^A, D^68^A) responsible for binding the complexin central helix also interferes with sytI-induced fusion in an in vitro fusion assay. While the authors’ interpretation that throwing the ‘switch’ lifts a fusion clamp on a neighboring SNARE complex is highly controversial (see above), changing the relative position of the accessory helix and N-terminus might still be essential for switching the mode of complexin action. Given that the very N-terminus is fulfilling a facilitating function, such conformational changes might place the domain close to the C-terminal end of the SNARE complex where it promotes full assembly of the SNARE proteins or regulates the binding configuration of sytI. In both scenarios, it might exert a fusion-promoting function wherein sytI and complexin act as allies affecting vesicle fusogenicity and triggering. A related view on complexin function was recently offered by Erwin Neher, who suggested that it may act as an allosteric adaptor for sytI [119]. Importantly, instead of postulating autonomous ‘clamp’ and ‘triggering’ functions, this interpretation explains the dual action of complexin as facets of a simple allosteric mechanism, by which complexin modulates the Ca^2+^-dependency of release. Following this line of argumentation, complexin loss may reduce the dynamic range of the Ca^2+^-dependent secretion by invoking changes in the energy levels of the Ca^2+^-bound (increased energy barrier) and its free states (decreased energy barrier).

In summary, current evidence points to clearly antagonistic roles of complexin and sytI in clamping, with Ca^2+^:sytI, possibly activating the arrested fusion intermediate without dislodging complexin (Fig. 2). The antagonistic action of both proteins might even carry on to very late stages of the fusion process, in which complexin restricts fusion pore expansion, while sytI promotes it. In facilitating release, sytI and complexin cooperate as partners, but the interdependency of their actions needs further investigation.

### Concluding remarks

Almost 20 years after its identification as an SNARE-interacting protein, complexin still remains an enigma. Even though it has become clear by now that complexin serves a dual function in vesicle fusion, namely clamping of premature release and facilitation of Ca^2+^-triggered vesicle fusion, the underlying molecular mechanisms are still far from understood. Evidently, complexin is small but capable, because it seems to affect crucial players in the exocytotic machinery with its few domains. Nevertheless, based on our current knowledge, it needs to be stressed that the seemingly counter-intuitive combination of fusion-inhibiting and fusion-promoting functions within one small accessory factor has a tremendous synergistic impact on the fidelity of Ca^2+^-triggered secretion.

## Abbreviations

AH: Accessory α-helix
EM: Electron micrograph
[Ca]i: Intracellular calcium
CH: Central helix
cplx: Complexin
CT: C-Terminus
hGH: Human growth hormone
PC12: Pheochromocytoma cell line
SER: Serine
SNAREs: *N*-Ethylmaleimide-sensitive factor (NSF) attachment protein receptors
SNAP25: Synaptosomal-associated protein, 25 kDa
sybII: Synaptobrevin II
stxIa: Syntaxin Ia
syt: Synaptotagmin
NT: N-Terminus
NMJ: Neuromuscular junction
